# Different effects of SGLT2 inhibitors according to the presence and types of heart failure in type 2 diabetic patients

**DOI:** 10.1186/s12933-020-01042-3

**Published:** 2020-05-28

**Authors:** In-Chang Hwang, Goo-Yeong Cho, Yeonyee E. Yoon, Jin Joo Park, Jun-Bean Park, Seung-Pyo Lee, Hyung-Kwan Kim, Yong-Jin Kim, Dae-Won Sohn

**Affiliations:** 1 Cardiovascular Center and Department of Internal Medicine, Seoul National University Bundang Hospital, Seongnam, Gyeonggi South Korea; 2 Department of Internal Medicine, Seoul National University College of Medicine, Seoul, South Korea; 3 Cardiovascular Center and Department of Internal Medicine, Seoul National University Hospital, Seoul, South Korea

**Keywords:** Heart failure, Diabetes, Echocardiography, Sodium-glucose cotransporter 2 inhibitor, SGLT2 inhibitor

## Abstract

**Background:**

The effects of sodium-glucose cotransporter 2 inhibitor (SGLT2i) on cardiac function are not fully understood. We investigated the changes in cardiac function in diabetic patients according to the presence and types of heart failure (HF).

**Methods:**

We retrospectively identified 202 diabetic patients who underwent echocardiography before, and 6 to 24 months after the initiation of SGLT2i. After propensity score matching with diabetic patients without SGLT2i, the study population (n = 304) were categorized into group 1 (without HF nor SGLT2i; n = 76), group 2 (without HF and received SGLT2i; n = 78), group 3 (with HF but without SGLT2i; n = 76), and group 4 (with HF and received SGLT2i; n = 74). Changes in echocardiographic parameters were compared between these 4 groups, and between HF patients with reduced versus preserved ejection fraction (EF).

**Results:**

After a median 13 months of follow-up, HF patients with SGLT2i showed a significant decrease in left ventricular end-diastolic dimension (LV-EDD; from 57.4 mm [50.0–64.9] to 53.0 mm [48.0–60.0]; p < 0.001) and improvement in LV-EF (from 36.1% [25.6–47.5] to 45.0% [34.8–56.3]; p < 0.001). LV mass index and diastolic parameters also showed improvements in HF patients with SGLT2i. The SGLT2i-induced improvements in cardiac function were more prominent in HF patients than those without HF, and in HFrEF patients than HFpEF patients.

**Conclusions:**

Use of SGLT2i improved cardiac function in diabetic patients, regardless of the presence of HF. The improvements were more prominent in HF patients, especially in those with HFrEF. These improvements in cardiac function would contribute to the clinical benefit of SGLT2i.

## Background

Sodium glucose co-transporter 2 (SGLT2) is the major cotransporter involved in glucose reabsorption in the kidney, and the inhibition of SGLT2 has been established as a novel therapeutic measure for diabetic control. In particular, the SGLT2 inhibitors (SGLT2i) have demonstrated robust benefits in terms of the prevention of heart failure (HF) and the reduction in cardiovascular mortality, which was the first evidence for prevention of major cardiovascular events by anti-diabetic medications. Several large-scale trials showed concordant results of SGLT2i for HF prevention [1–3].

Adding to the glucose lowering effect of SGLT2i, several glucose-independent mechanisms have been suggested for HF prevention: preload and afterload reduction by natriuresis and osmotic diuresis, improvement in cardiac metabolism and bioenergetics, reduced myocardial necrosis and fibrosis, and alteration in adipokines [4]. These hypotheses were further supported by the recently published trial of dapagliflozin, the Dapagliflozin and Prevention of Adverse-outcomes in Heart Failure (DAPA-HF) trial, which extended the benefits of SGLT2i to those with HF with reduced ejection fraction (HFrEF) even in the absence of diabetes.

Despite the well-established clinical benefits of SGLT2i, only limited studies on the changes in cardiac function by SGLT2i have been made so far. Furthermore, it is unclear whether the cardiovascular benefits of SGLT2i would be similar between the diabetic patients with versus without HF, and between those with HFrEF versus HFpEF. We aimed to compare the SGLT2i-induced changes in cardiac function and geometry assessed by echocardiography in type 2 diabetic patients according to the presence and types of HF, using a retrospective cohort.

## Methods

### Patients

This retrospective (cohort) study was carried out according to the principles of the Declaration of Helsinki and was approved by the Clinical Research Institute of Seoul National University Bundang Hospital (approved on March 11 2019; IRB No. B-1903-531-105).

**Fig. 1:**
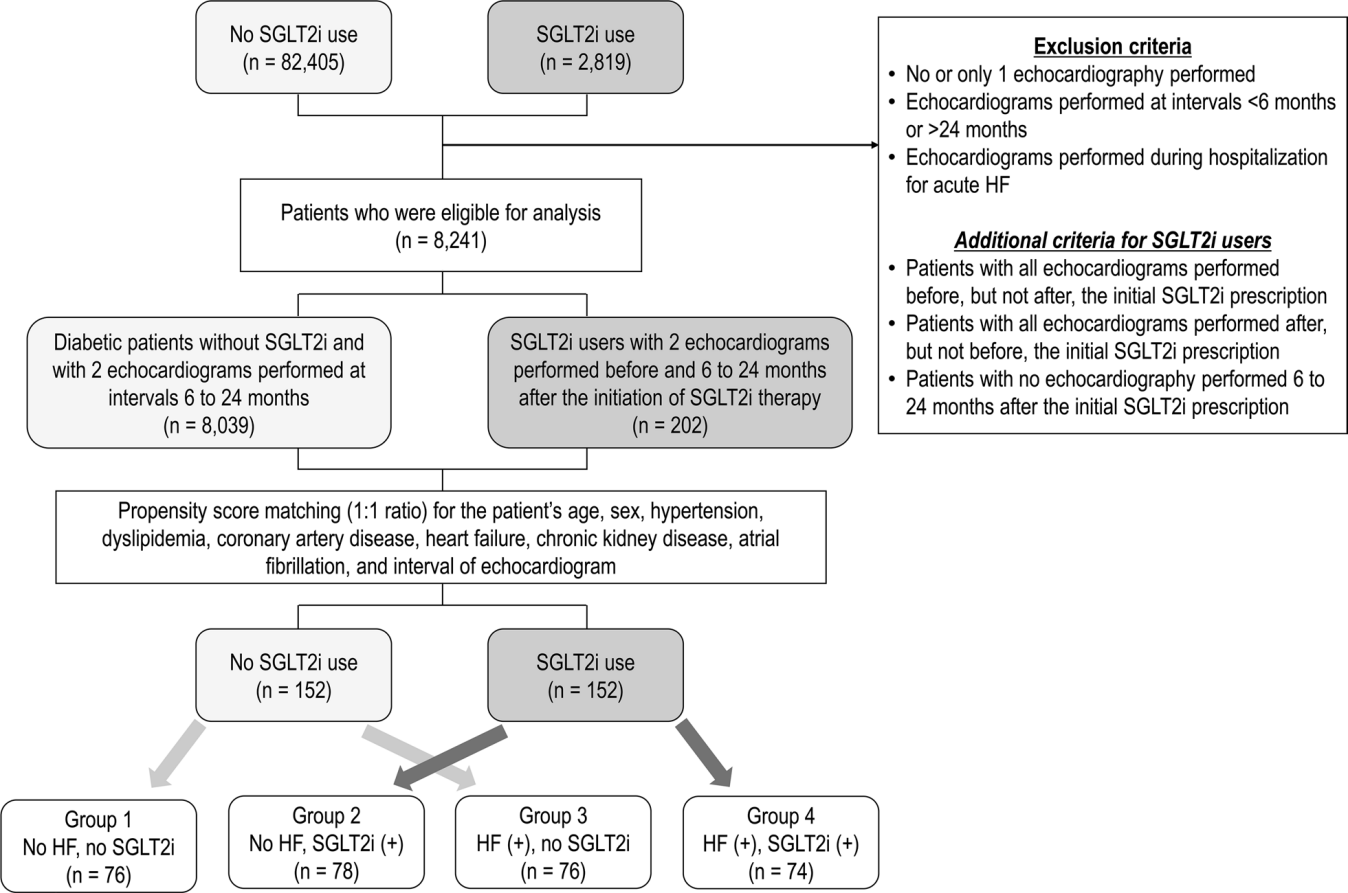
Flow chart of study population. *SGLT2i* sodium-glucose cotransporter 2 inhibitor, *HF* heart failure

From October 2014 to April 2019, we retrospectively identified 2819 patients with type 2 diabetes who visited Seoul National University Bundang Hospital and were prescribed with SGLT2i (1679 patients prescribed with dapagliflozin; 1131 with empagliflozin; and 9 with ipragliflozin) (Fig. 1). Comorbidities of patients were determined from the electronic medical record. Patients without echocardiography or with only 1 echocardiography were excluded. Among the patients with 2 or more echocardiograms, those with the interval of two echocardiograms < 6 months or > 24 months were excluded. Patients for whom echocardiograms were performed during hospitalization for acute HF were also excluded. Using these exclusion criteria, 8039 diabetic patients without SGLT2i prescription were identified.

For the patients with SGLT2i prescription, those with all echocardiograms performed before, but not after, the initial SGLT2i prescription were excluded. Patients with all echocardiograms performed after, but not before, the initial SGLT2i prescriptions were also excluded. In addition, patients with no echocardiography performed 6–24 months after the initial SGLT2i prescription were excluded. After exclusion, 202 diabetic patients for whom SGLT2i were prescribed, and for whom the baseline echocardiography was performed before the initial SGLT2i prescription and the follow-up echocardiography was performed at 6–24 months after the initial SGLT2i prescription were identified.

Using the propensity score matching with 1:1 ratio for age, sex, hypertension, dyslipidemia, coronary artery disease, HF, chronic kidney disease, atrial fibrillation, and interval of echocardiogram, the 202 diabetic patients with SGLT2i prescription were matched to 8039 diabetic patients without SGLT2i (Additional file 1: Table S1). The definition of coronary artery disease was a presence of ≥ 50% diameter-stenosis on invasive coronary angiography or computed tomography angiography, a presence of perfusion decrease on myocardial perfusion imaging, or a positive result on treadmill test. HF was defined as the presence of New York Heart Association functional class ≥ II and elevated N-terminal pro-B-type natriuretic peptide (NT-proBNP; > 125 pg/mL) or echocardiographic findings suggestive of HF (left ventricular ejection fraction [LV-EF] < 40%, diastolic dysfunction, or other relevant structural heart disease including LV hypertrophy and/or left atrial enlargement), according to the clinical guidelines [5, 6]. The chronic kidney disease was defined as the glomerular filtration rate < 60 mL/min/1.73 m^2^.

The propensity score matching was successful, and a total of 152 diabetic patients on SGLT2i and a total of 152 diabetic patients without SGLT2i were identified (Fig. 1). We categorized the study population into 4 groups divided according to the presence of HF and the prescription of SGLT2i: diabetic patients without HF and without SGLT2i (group 1; n = 76), diabetic patients without HF and with SGLT2i (group 2; n = 78), diabetic patients with HF but without SGLT2i (group 3; n = 76), and diabetic patients with HF and with SGLT2i (group 4; n = 74).

### Echocardiography and strain analysis

Results of the baseline and follow-up echocardiography were obtained from the database. Standard techniques were used to obtain M-mode, 2-dimensional, and Doppler measurements in accordance with the guidelines [7]. The LV global longitudinal strain (LV-GLS) measurements were performed by experienced echocardiography specialists in a blinded fashion using TomTec software (Image Arena 4.6, Munich, Germany), according to current guidelines [8]. We took the absolute value |x| of LV-GLS for simpler interpretation.

### Study outcomes

The study outcomes were the changes in echocardiographic parameters of LV geometry (LV end-diastolic dimension [LV-EDD], LV end-diastolic volume [LV-EDV], and LV mass index [LV-MI]), systolic function (LV-EF and LV-GLS), and diastolic function (mitral E/e’ ratio, and the estimated pulmonary artery systolic pressure [PASP]).

The changes in echocardiographic parameters were compared between the four subgroups divided according to the presence of HF and the SGLT2i prescription. In addition, as a sensitivity analysis, we compared the changes in echocardiographic parameters according to the use of SGLT2i between patients with HF with reduced EF (LV-EF < 40% at baseline) and those with HF with preserved EF (HFpEF) (LV-EF ≥ 40% at baseline).

### Statistical analysis

Categorical variables are presented as frequencies and percentages, and continuous variables as means ± standard deviations (SD) or medians with interquartile ranges (IQR). Group comparisons were performed with student *t* test or paired t-test for normally distributed data and the Wilcoxon matched pairs test when data were non-normal. The χ^2^ test or the Fisher’s exact test was used for categorical variables. In order to assess the association between the use of SGLT2i and the improvement LV-EF, we used logistic regression analysis for the +5% improvement in LV-EF and for the +10% improvement in LV-EF. Multivariable logistic regression analysis with stepwise backward elimination method was performed all variables of p-values < 0.20 by univariate analyses. All of the statistical analyses were performed using SPSS 21.0 (SPSS Inc, Chicago, Ill) and a p-value < 0.05 was considered statistically significant.

## Results

### Baseline characteristics and candidate predictors

In total study population, the median age was 66 years (56–74), and 65% were male (Table 1). Prevalence of comorbidities were not different across the subgroups. Eighty-two percent of patients were on metformin, 60% were on sulfonylurea, and 69% were on DPP-4 inhibitors. The levels of fasting serum glucose and HbA1c were not different across the subgroups. The median follow-up interval of the total study population was 13 (8–20) months, and was not different between the subgroups.

**Table 1:** Baseline characteristics

	Total study population (n = 304)	Group 1 No HF, no SGLT2i (n = 76)	Group 2 No HF, SGLT2i (+) (n = 78)	p-value (groups 1 vs. 2)	Group 3 HF (+), no SGLT2i (n = 76)	Group 4 HF (+), SGLT2i (+) (n = 74)	p-value (groups 3 vs. 4)
Age (years)	66 (56–74)	66 (58–72)	65 (56–72)	0.470	67 (56–75)	67 (54–75)	0.950
Male sex (n, %)	198 (65.1%)	44 (57.9%)	52 (66.7%)	0.261	50 (65.8%)	52 (70.3%)	0.676
Systolic blood pressure (mmHg)	132 (118–148)	136 (119–148)	134 (121–154)	0.832	126 (113–148)	130 (116–143)	0.924
Diastolic blood pressure (mmHg)	76 (68–85)	75 (70–81)	78 (65–88)	0.876	78 (68–85)	76 (68–84)	0.800
Heart rate (per minute)	79 (68–91)	76 (66–87)	75 (68–83)	0.638	83 (68–99)	83 (71–97)	0.635
NYHA functional class III/IV	53 (17.4%)	0 (0.0%)	0 (0.0%)	N/A	26 (34.2%)	27 (36.5%)	0.771
Hypertension	149 (49.9%)	37 (48.7%)	31 (39.7%)	0.264	43 (56.6%)	38 (51.4%)	0.631
Dyslipidemia	101 (33.2%)	25 (32.9%)	23 (29.5%)	0.648	27 (35.5%)	26 (35.1%)	0.960
Coronary artery disease	110 (36.2%)	26 (34.2%)	31 (39.7%)	0.477	27 (35.5%)	26 (35.1%)	0.828
Chronic kidney disease	36 (11.8%)	10 (13.2%)	8 (10.3%)	0.575	9 (11.8%)	9 (12.2%)	0.952
Atrial fibrillation	96 (31.6%)	13 (17.1%)	14 (17.9%)	0.891	36 (47.4%)	33 (44.6%)	0.858
Medications							
Use of SGLT2i							
Dapagliflozin	69 (22.7%)	0 (0.0%)	39 (50.0%)	N/A	0 (0.0%)	29 (39.2%)	N/A
Empagliflozin	83 (27.3%)	0 (0.0%)	39 (50.0%)	N/A	0 (0.0%)	45 (60.8%)	N/A
Duration of SGLT2i use (months)	–	–	10 (5–15)	N/A	–	10 (7–15)	N/A
Statin	254 (83.6%)	69 (90.8%)	68 (87.2%)	0.475	58 (76.3%)	59 (79.7%)	0.614
Metformin	249 (81.9%)	59 (77.6%)	63 (80.8%)	0.631	61 (80.3%)	66 (89.2%)	0.174
Sulfonylurea	184 (60.5%)	38 (50.0%)	50 (64.1%)	0.077	45 (59.2%)	51 (68.9%)	0.216
DPP-4 inhibitors	210 (69.1%)	47 (61.8%)	56 (71.8%)	0.189	55 (72.4%)	52 (70.3%)	0.776
ACE inhibitors	107 (35.2%)	20 (26.3%)	26 (33.3%)	0.341	29 (38.2%)	32 (43.2%)	0.526
ARB	161 (53.0%)	41 (53.9%)	38 (48.7%)	0.516	42 (55.3%)	40 (54.1%)	0.882
Beta-blockers	255 (83.9%)	58 (76.3%)	61 (78.2%)	0.780	67 (88.2%)	69 (93.2%)	0.284
MRA	103 (33.9%)	9 (11.8%)	9 (11.5%)	0.953	40 (52.6%)	45 (60.8%)	0.312
Diuretics	179 (58.9%)	23 (30.3%)	31 (39.7%)	0.218	59 (77.6%)	66 (89.2%)	0.079
Baseline laboratory tests							
Hemoglobin (g/dL)	13.8 (12.7–15.1)	13.7 (12.8–15.0)	14.1 (13.1–15.0)	0.368	13.7 (12.3–15.3)	13.6 (12.1–15.3)	0.624
Serum creatinine (mg/dL)	0.9 (0.8–1.1)	0.9 (0.7–1.1)	0.9 (0.8–1.1)	0.839	1.0 (0.8–1.2)	1.0 (0.8–1.3)	0.806
Total cholesterol (mg/dL)	144.5 (122.5–172.0)	151.5 (127.0–174.0)	142.5 (119.0–172.0)	0.171	136.0 (119.0–169.0)	142.5 (120.0–170.0)	0.930
Fasting glucose (mg/dL)	136.0 (117.0–159.0)	138.0 (119.0–161.5)	138.0 (116.0–175.0)	0.427	129.5 (113.5–149.5)	132.0 (117.0–155.0)	0.363
HbA1c (%)	7.1 (6.5–8.0)	7.3 (6.6–7.8)	7.5 (6.6–8.6)	0.095	6.8 (6.4–7.8)	6.9 (6.4–7.7)	0.905
NT-proBNP (pg/mL)	447.7 (106.9–2432.6) (n = 207)	100.0 (54.8–199.2) (n = 30)	77.3 (32.7–197.8) (n = 36)	0.858	925.6 (311.9–3386.1) (n = 70)	1819.6 (547.3–5695.4) (n = 71)	0.637
Echocardiographic parameters							
LV-EDV (mL)	92.0 (69.0–128.5)	76.0 (64.5–96.5)	79.5 (65.0–97.0)	0.430	111.9 (82.5–148.5)	128.5 (94.0–169.0)	0.098
LV-ESV (mL)	41.0 (28.9–80.7)	29.6 (23.5–42.8)	32.0 (25.0–42.0)	0.486	69.5 (33.0–112.0)	82.5 (41.0–125.0)	0.115
LV-EDD (mm)	50.9 (46.5–80.0)	48.0 (44.5–50.0)	49.0 (45.0–52.0)	0.174	56.1 (49.5–62.0)	57.4 (50.0–64.9)	0.203
LV-ESD (mm)	35.7 (30.0–47.0)	31.0 (28.4–35.5)	32.0 (28.0–36.0)	0.658	44.0 (32.5–51.0)	45.4 (37.0–54.0)	0.181
LV-EF (%)	52.0 (35.6–61.1)	60.4 (52.4–63.8)	59.4 (49.3–63.6)	0.628	38.8 (28.0–55.9)	36.1 (25.6–47.5)	0.094
LV-MI (g/m^2^)	112.8 (91.0–134.7)	99.6 (84.1–123.3)	96.6 (82.7–114.7)	0.613	120.9 (101.3–146.5)	126.3 (111.1–147.3)	0.666
LAVI (mL/m^2^)	40.3 (31.0–56.5)	31.8 (28.3–39.9)	34.7 (29.5–44.8)	0.081	48.2 (36.2–68.1)	53.5 (41.6–72.3)	0.860
PASP (mmHg)	28.0 (24.4–38.6)	26.2 (23.0–29.3)	26.2 (22.6–30.0)	0.808	32.0 (26.2–41.0)	36.4 (28.0–54.0)	0.038
E velocity (m/s)	0.70 (0.58–0.87)	0.66 (0.59–0.78)	0.63 (0.57–0.78)	0.760	0.73 (0.58–0.87)	0.79 (0.63–1.03)	0.030
Mitral annular e′ velocity (cm/s)	5.7 (4.2–7.2)	6.10 (4.80–7.40)	6.00 (5.00–7.10)	0.403	5.7 (4.2–7.2)	5.10 (3.70–6.50)	0.167
Mitral annular s’ velocity (cm/s)	6.0 (4.7–7.5)	6.7 (5.4–8.4)	6.6 (5.5–7.8)	0.696	5.3 (4.2–6.7)	5.0 (3.8–6.1)	0.377
Mitral E/e′ ratio	12.6 (9.3–17.1)	10.8 (8.9–14.1)	10.6 (9.0–13.5)	0.742	13.2 (9.8–17.8)	15.6 (11.9–24.3)	0.026
LV-GLS (%)	12.5 (9.5–15.5)	15.2 (12.5–16.9)	14.6 (12.1–17.0)	0.962	10.9 (8.4–12.3)	10.3 (7.3–12.5)	0.532
Follow-up interval (months)	13 (8–20)	13 (8–21)	12 (9–19)	0.091	11 (7–16)	14 (8–21)	0.183

Data are expressed as median with interquartile range (Q1–Q3) or as number (percentage)

*HF* heart failure, *SGLT2i* sodium-glucose cotransporter 2 inhibitor, *NYHA* New York Heart Association, *DPP-4* dipeptidyl peptidase-4, *ACEi* angiotensin converting enzyme inhibitor, *ARB* angiotensin receptor blocker, *MRA* mineralocorticoid antagonist, *HbA1c* hemoglobin A1c, *LV* left ventricular, *EDV* end-diastolic volume, *ESV* end-systolic volume, *EDD* end-diastolic dimension, *ESD* end-systolic dimension, *EF* ejection fraction, *MI* mass index, *LAVI* left atrial volume index, *PASP* pulmonary artery systolic pressure, *GLS* global longitudinal strain

The types and doses of SGLT2i were not different between group 2 vs. 4 (Table 1 and Additional file 1: Table S2). Eighty-three patients (27.3%) were on empagliflozin, and 69 (22.7%) on dapagliflozin. The duration of SGLT2i use was 10 (5–15) months in group 2, and was 10 (7–15) months in group 4. Use of angiotensin converting enzyme inhibitors (ACEi), angiotensin receptor blocker (ARB), beta-blockers, mineralocorticoid antagonists (MRA), and diuretics were more frequent in patients with HF (groups 3 and 4) compared to those without HF (groups 1 and 2), but were not different according to the use of SGLT2i (groups 1 vs. 2; groups 3 vs. 4). Also, the types of cardioprotective medications (ACEi, ARB, beta-blockers, and MRA) and their changes during follow-up were not different according to the use of SGLT2i. Detailed information on the use of medications are summarized in Additional file 1: Table S2.

### Comparison of echocardiographic parameters according to the presence of HF

At baseline, the patients with HF (groups 3 and 4) had worse echocardiographic parameters compared to those without HF (groups 1 and 2), but there was no difference according to the use of SGLT2i, both in those without HF (groups 1 vs. 2) and in those with HF (groups 3 vs. 4) (Table 1).

**Fig. 2:**
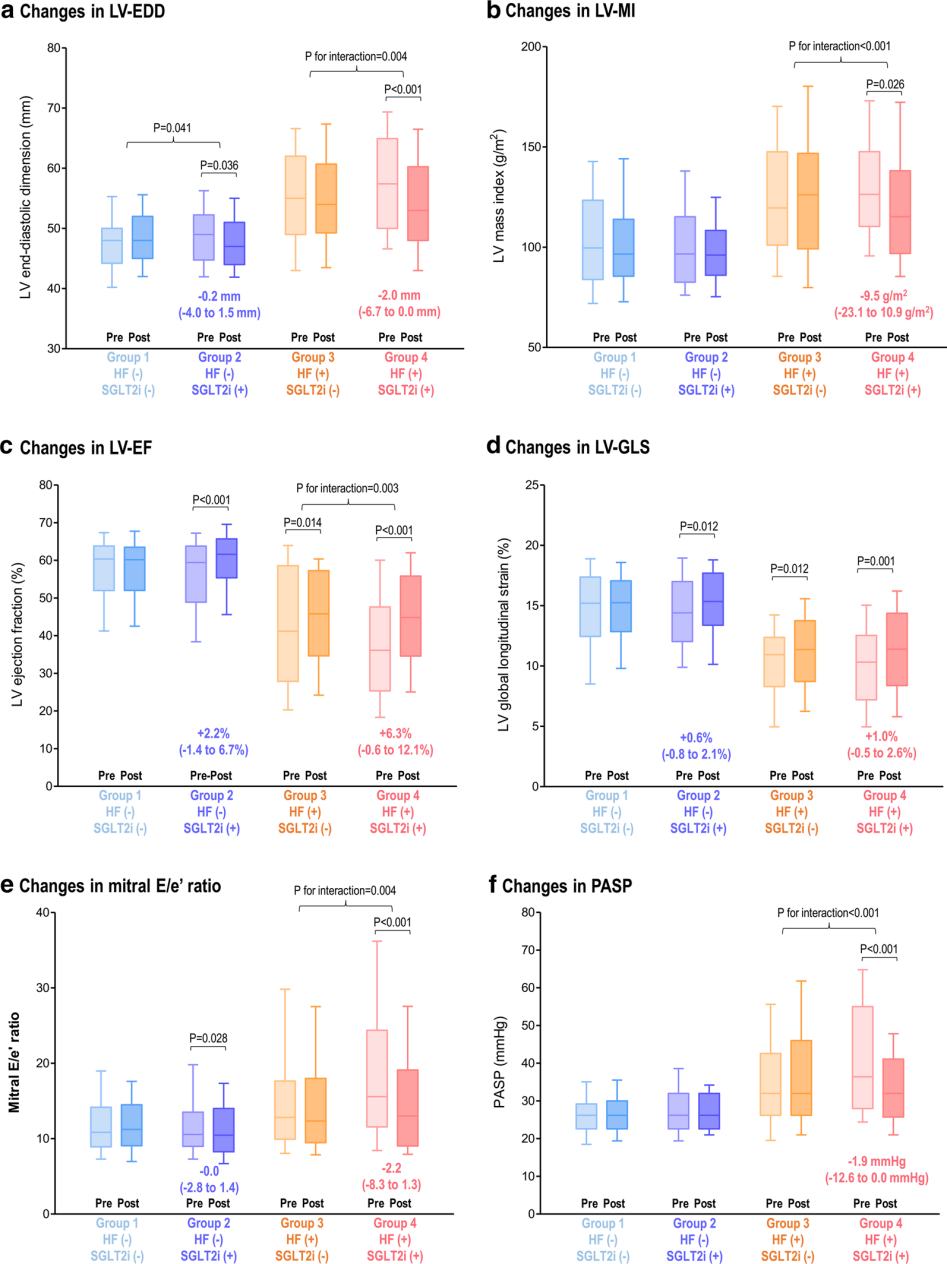
Changes in LV function and geometry by SGLT2i according to the presence of HF and the use of SGLT2i. Echocardiographic parameters at baseline and follow-up are presented according to the presence of HF and the use of SGLT2i: **a** LV-EDD, **b** LV-MI, **c** LV-EF, **d** LV-GLS, **e** mitral E/e′ ratio, and **f** PASP. Bars represent the median with interquartile range (Q1–Q3). Intra-group and inter-group comparisons were performed with paired t-test generalized linear model for repeated measure analysis, respectively. *LV* left ventricular, *EF* ejection fraction, *GLS* global longitudinal strain, *EDD* end-diastolic dimension, *MI* mass index, *PASP* pulmonary arterial systolic pressure; others as in Fig. 1

At follow-up, the LV geometry showed significant differences according to the use of SGLT2i and to the presence of HF (Fig. 2). In patients without HF, those with SGLT2i treatment showed a small but significant decrease in LV-EDD (from 49.0 [45.0–52.0] to 47.0 mm [44.0–51.0]; p = 0.036), but those without SGLT2i did not (p for interaction = 0.041) (Fig. 2a). In the HF patients, we observed a significant decrease in LV-EDD (from 57.4 [50.0–64.9] to 53.0 mm [48.0–60.0]; p < 0.001), as well as LV-MI (from 126.3 [111.1–147.3] to 115.2 g/m^2^ [97.0–137.8]; p = 0.026) in SGLT2i treated group, but not in the HF patients without SGLT2i treatment (p for interaction = 0.004 for LV-EDD, < 0.001 for LV-MI) (Fig. 2a, b).

In patients without HF, the SGLT2i users showed a significant increase in LVEF in group 2 (from 59.4 [49.3–63.6] to 61.6% [55.6–65.7]; p < 0.001) but not in the group 1 (from 60.4 [52.4–63.8] to 60.2% [52.1–63.3]) (Fig. 2c). Similarly, the LV-GLS was improved in group 2 but not in group 1 (Fig. 2d). In the HF patients, those with SGLT2i treatment (group 4) showed a prominent improvement in LV-EF (from 36.1 [25.6–47.5] to 45.0% [34.8–56.3]; p < 0.001) as well as LV-GLS (from 10.3 [7.3–12.5] to 11.4% [8.4–14.3]; p = 0.001). The HF patients without SGLT2i (group 3) also demonstrated subtle improvement in LV-EF and LV-GLS, but the improvement in LV-EF was more prominent in the HF patients with SGLT2i treatment (group 4) (p for interaction = 0.003 for LV-EF). Multivariable logistic regression analysis showed that the use of SGLT2i was significantly associated with +5% improvement in LV-EF (adjusted OR, 2.384; 95% CI, 1.266–4.448; p = 0.007) (Table 2 and Additional file 1: Table S3). For +10% improvement in LV-EF, the use of SGLT2i (adjusted OR, 2.236; 95% CI, 1.106–4.521; p = 0.025) and the standard dose of beta-blockers for HF (adjusted OR, 2.849; 95% CI, 1.126–7.205; p = 0.027) showed significant associations (Table 2 and Additional file 1: Table S3). We further assessed the changes in LV diastolic function parameters (Fig. 2). A reduction in mitral E/e’ ratio was observed in the group 2 (from 10.6 [9.0–13.5] to 10.5 [8.3–14.0]; p = 0.028) and group 4 (from 15.6 [11.9–24.3] to 13.0 [9.1–19.1]; p < 0.001), but not observed in those without SGLT2i treatment (groups 1 and 3) (Fig. 2e). The group 4 patients also showed a significant decrease in PASP (from 36.4 [28.0–54.0] to 32.0 mmHg [26.2–41.0]; p < 0.001), but not in the group 3 patients (p for interaction < 0.001) (Fig. 2f).

**Table 2:** Multivariate analysis for the improvement in LV-EF

	+ 5% improvement in LV-EF			+ 10% improvement in LV-EF		
	Adjusted OR	95% CI	p-value	Adjusted OR	95% CI	p-value
Age (per +1 year)	0.965	0.937–0.993	0.015	0.975	0.949–1.002	0.070
Male sex	–	–	–	0.271	0.125–0.584	0.001
Hypertension	0.521	0.278–0.975	0.042	–	–	–
SGLT2i	2.384	1.266–4.488	0.007	2.236	1.106–4.521	0.025
Standard dose of beta-blockers for HF^a^	–	–	–	2.849	1.126–7.205	0.027
LV-EDV (per +1 mL)	0.987	0.977–0.996	0.007	–	–	–
LV-EF (per +1%)	0.919	0.891–0.948	< 0.001	0.928	0.906–0.950	< 0.001
PASP (per +1 mmHg)	1.025	1.000–1.051	0.049	–	–	–

Multivariate logistic regression analysis was performed in the total study population. Univariate factors with p-values < 0.200 entered the multivariate analysis, using stepwise backward elimination methods to select the factors for inclusion in the multivariable analysis

*LV* left ventricular, *EF* ejection fraction, *OR* odds ratio, *CI* confidence interval, *HTN* hypertension, *SGLT2i* sodium-glucose cotransporter 2 inhibitor, *EDV* end-diastolic volume, *PASP* pulmonary artery systolic pressure

^a^Standard doses of beta-blockers for HF were determined according to the 2016 ESC Guidelines for the diagnosis and treatment of acute and chronic heart failure [6]

There was no difference in the changes in echocardiographic parameters according to the type of SGLT2i (dapagliflozin vs. empagliflozin; Additional file 1: Figures S1 and S2).

### Comparison of LV function changes between HFrEF and HFpEF

**Fig. 3:**
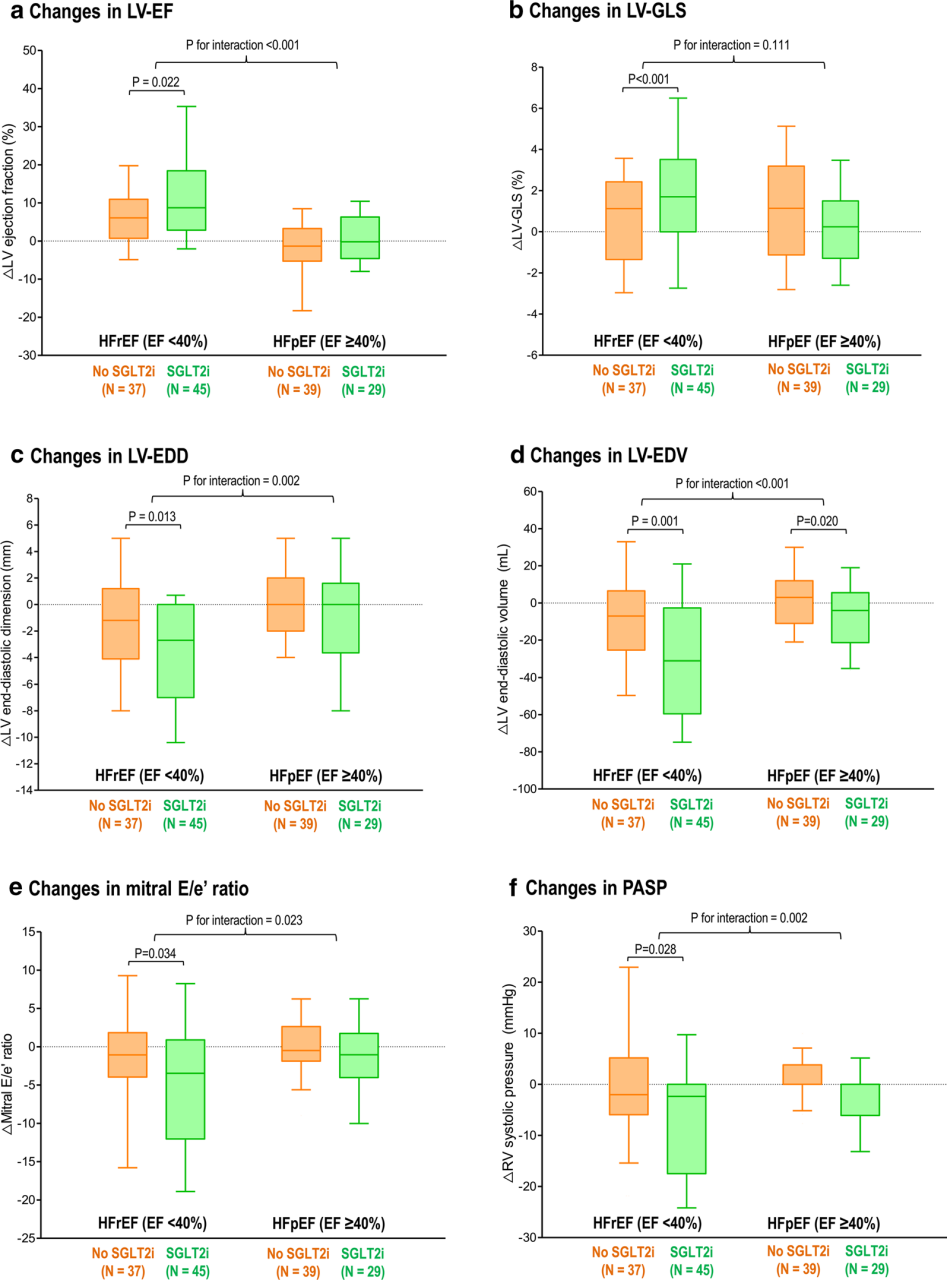
Changes in LV function and geometry by SGLT2i according to the types of HF. The changes in echocardiographic parameters were compared between subgroups divided according to the types of HF (HFrEF vs. HFpEF) and the use of SGLT2i: **a** LV-EF, **b** LV-GLS, **c** LV-EDD, **d** LV-EDV, **e** mitral E/e′ ratio, and **f** PASP. Bars represent the median with interquartile range (Q1–Q3). Intra-group and inter-group comparisons were performed with paired t-test generalized linear model for repeated measure analysis, respectively. Abbreviations as in Figs. 1 and 2

In patients with HFrEF (LV-EF < 40%), the LV systolic function parameters showed significant improvement in those with SGLT2i treatment (ΔLV-EF, +8.8% [3.0–17.9]; ΔLV-GLS, +1.7% [0.1–3.5]), but not in those without SGLT2i treatment (p < 0.001 for both LV-EF and LV-GLS) (Fig. 3). Of note, the changes in LV-EF and LV-GLS did not show different according to the SGLT2i treatment in HFpEF patients.

Similar patterns were observed for LV geometry and diastolic function. The HFrEF patients with SGLT2i treatment showed significant decreases in LV-EDD, LV-EDV, mitral E/e’ ratio and PASP, whereas the improvements in LV geometry and diastolic function were not observed in the HFpEF patients with SGLT2i treatment (Fig. 3).

### Changes in NT-proBNP levels

**Fig. 4:**
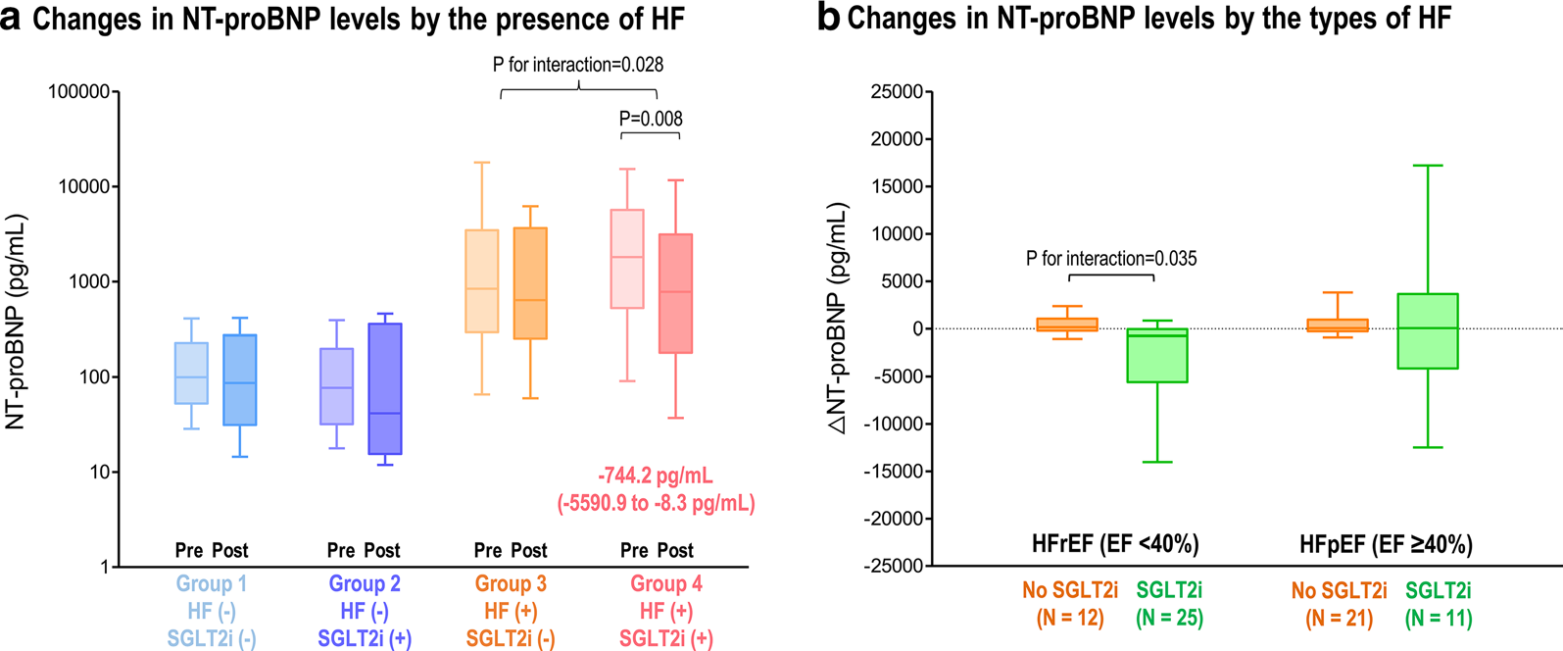
Changes in NT-proBNP levels by SGLT2i according to the presence and types of HF. The changes in the levels of NT-proBNP were compared according to the use of SGLT2i and the presence of HF (**a**), and the types of HF (**b**). Abbreviations: as in Figs. 1 and 2

We compared the changes in NT-proBNP levels in patients with both baseline and follow-up measurement (n = 76) (Fig. 4a). The HF patients with SGLT2i treatment (group 4) showed a significant decrease in NT-proBNP levels (from 1819.6 [547.3–5695.4] to 782.1 pg/mL [181.5–2954.0]; p = 0.008), but other groups did not (p for interaction = 0.028 between group 4 vs. 3).

Among the HF patients with both baseline and follow-up NT-proBNP measurements (n = 69), the reduction in NT-proBNP was observed only in HFrEF patients treated with SGLT2i (from 2202.5 [572.4–6131.4] to 767.5 pg/mL [183.1–1688.7]; p = 0.001), but not in the HFrEF patients without SGLT2i (p for interaction = 0.035 between HFrEF with SGLT2i vs. HFrEF without SGLT2i) (Fig. 4b). The HFpEF patients did not show significant changes in NT-proBNP levels regardless of the use of SGLT2i.

## Discussion

In this retrospective cohort study, we observed that the use of SGLT2i was associated with improvements in LV geometry, systolic and diastolic function in type 2 diabetic patients. The improvement in cardiac function by SGLT2i was more prominent in HF patients, compared to the patients without HF. Of note, among the diabetic patients with HF, the improvement in cardiac function was observed in HFrEF patients, but not in HFpEF patients. Our findings provide background for the clinical benefits reported in recent trials, and suggest that the benefits of SGLT2i in HF patients may be more prominent in HFrEF, compared to HFpEF.

### Established cardiovascular benefits of SGLT2i and potential mechanisms

The SGLT2i showed robust evidence for prevention of HF and cardiovascular mortality: a meta-analysis of large-scale trials showed that the use of SGLT2i leads to about 30% reduction in HF hospitalization and cardiovascular mortality in HF patients, and about 20% reduction in HF hospitalization and cardiovascular mortality in diabetic patients without HF [1–3, 9]. Because these benefits appeared at early phase of the clinical trials, which cannot be explained by the glucose lowering effect, multiple glucose-independent mechanisms have been suggested. Most of all, the diuretic effect of SGLT2i would be the key component for HF prevention which is different from those observed with conventional diuretics: (1) SGLT2i do not affect plasma osmolarity (2) activates the tubule-glomerular feedback through the increased delivery of fluid and electrolytes to the macula densa, (3) produces a greater fluid clearance from the interstitial space resulting in a better congestion relief with minimal impact on arterial filling and organ perfusion, and (4) exerts renoprotective effects [4, 10, 11].

In addition, the SGLT2i may provide cardiovascular benefits by an improved myocardial energy metabolism [4, 12]. The use of SGLT2i results in hyperketonemia, and switches myocardial fuel utilization from glucose to ketone bodies and free fatty acid, resulting in a more efficient ATP production [13, 14]. Also, the SGLT2i can inhibit cardiac Na^+^-H^+^ exchanger (NHE), resulting in the reduction of intracellular calcium and an increase of mitochondrial Ca^2+^, which restores mitochondrial function and redox state, activates ATP production [15]. These potential mechanistic background was further supported by animal model studies: the use of SGLT2i attenuated myocardial oxidative stress and fibrosis in diabetic mice heart, and improved coronary microvascular function and cardiac contractility in pre-diabetic mice model [16, 17].

Despite the absence of overt structural heart disease or symptoms of HF, the diabetic patients are considered as “stage A HF” because these patients are at high risk for HF [5, 18]. These patients often demonstrate subclinical myocardial dysfunction, which can be detected by diastolic dysfunction and LV hypertrophy [18, 19]. Furthermore, recent studies showed that impaired LV-GLS is a sensitive marker in diabetic patients with stage A HF [20, 21]. In our study, patients without overt HF can be considered as having stages A or B HF due to the presence of diabetes and the impaired LV-GLS values at baseline (LV-GLS 15.2% in group 1 and 14.6% in group 2), despite the preserved LV-EF. Given the high risk of HF development as well as the presence of subclinical LV dysfunction, there is a need for effective prevention of LV function deterioration in these patients. Several studies that showed improvements in LV diastolic function and reduction in LV-MI by SGLT2i in diabetic patients without overt HF [22, 23]. Also, a reduction in LV-MI in diabetic patients with coronary artery disease was observed in the Effects of Empagliflozin on Cardiac Structure in Patients with Type 2 Diabetes (EMPAHEART) CardioLink-6 trial [24]. Our findings are consistent with these studies that the use of SGLT2i resulted in small but significant improvements in LV geometry and diastolic function in diabetic patients without overt HF. Adding to this, we observed significant improvements in LV-EF and LV-GLS in diabetic patients without overt HF. These structural changes support the mechanistic background of SGLT2i in terms of the improved myocardial intrinsic function and energetics, and also explain the clinical benefits of SGLT2i for prevention of HF in diabetic patients even at the early stage without structural heart disease or HF symptoms.

### Cardioprotective effects of SGLT2i in patients with HF

The glucose-independent benefits of SGLT2i have been confirmed by recently published clinical trials: according to the DAPA-HF trial, the use of dapagliflozin reduced the composite of worsening HF or cardiovascular death to 27% in HFrEF patients, and the results were not different between those with diabetes and those without [25]. Also, the Dapagliflozin Effects on Biomarkers, Symptoms and Functional Status in Patients with HF with Reduced Ejection Fraction (DEFINE-HF) trial reported clinically meaningful improvements in HF-related health status or natriuretic peptides in HF patients regardless of the presence of diabetes [26]. Therefore, it can be reasonable to assume that the cardiovascular benefits of SGLT2i would be independent to its glucose-lowering effect, as demonstrated in various non-diabetic animal models. Byrne et al. investigated non-diabetic mice subjected to pressure overload and found that the 2-week of empagliflozin treatment preserved LV systolic function, which was sustained ex vivo in the absence of ketones or hemodynamic changes [27]. In a rat model of hypertensive HF, empagliflozin improved LV systolic function, reduced LV cavity size, and attenuated cardiac fibrosis [28]. Recently, Zhang et al. administered dapagliflozin to a pig model of HFpEF and observed that dapagliflozin reduced blood pressure, mitigated LV concentric remodeling, and attenuated hypertension-induced macrovascular inflammatory response [29].

In the present study, we observed more prominent improvements in LV function by SGLT2i in diabetic patients with HF compared to those without, which was independent of the use of cardioprotective medications for HF (ACEi, ARB, beta-blockers, and MRA). Many patients with HF (groups 3 and 4) had uptitration of HF medications during follow-up, and reached the standard dose for optimal HF management. Indeed, we observed that the use of standard dose of beta-blockers for HF was associated with an improved LV-EF among the diabetic patients with HF. More importantly, the use of cardioprotective medications and their changes were not different between those treated with SGLT2i and those without (groups 3 vs. 4), and the improvement of LV function by SGLT2i use was observed even after adjusting for the use of cardioprotective medications and their changes.

### Differences in SGLT2i effects between HFrEF vs. HFpEF

Several studies showed the changes in LV function parameters in diabetic patients according to the types of HF. A retrospective echocardiographic study of diabetic patients on either SGLT2i or DPP-4 inhibitors reported reductions in E/e’ ratio and NT-proBNP levels by SGLT2i in patients with LV-EF < 40%, but not in those with LV-EF ≥ 40% [30]. However, beneficial effect of SGLT2i has also been suggested in patients with HFpEF: in an echocardiographic study by Soga et al. the mitral E/e’ ratio, LV-MI, and LAVI were decreased with dapagliflozin treatment among diabetic patients with high prevalence of HFpEF (69%) and HFmrEF (17%), whereas the improvement in LV systolic function and the reduction in BNP levels were minimal [31]. A recent prospective study of diabetic patients with HFpEF demonstrated reductions in E/e’ ratio and BNP level, along with improved renal function, decreased fat volumes and reduced oxidative stress [32].

In the present study, we advanced to the comparison of echocardiographic changes by SGLT2i in diabetic patients according to the presence and types of HF. The improvements in LV function and geometry were more prominent in HF patients than in those with HF, and these improvements were mainly derived in the HFrEF patients rather than those with HFpEF. Of note, the reduction in NT-proBNP levels was observed only in those with HFrEF patients, in whom the baseline NT-proBNP levels were significantly higher compared to the HFpEF patients. These findings are in line with the study by Soga et al. where the levels of BNP were decrease by SGLT2i among those with baseline BNP levels ≥ 100 pg/mL [31]. These findings suggest that the improvement in cardiac function by SGLT2i would be attributable to the effective volume reduction and possibly to the alleviation of activated neurohormonal axis.

Based on these findings, we suppose that the action mechanisms of SGLT2i can provide possible explanation on the different echocardiographic responses between HFrEF and HFpEF. The levels of natriuretic peptide are higher in HFrEF than in HFpEF, due to the further stretched LV by volume overload and accompanied neurohormonal activation [33, 34]. Several network analysis studies of multiple biomarkers showed that the elevated NT-proBNP is one of the hub of HFrEF pathophysiology [33, 35], together with the activated biological pathways for cellular proliferation and cardiac hypertrophy [35]. In contrast, the biomarker studies showed that the main pathophysiology of HFpEF is the inflammation, integrin signaling, and extracellular matrix organization, rather than myocardial stretching characterized by elevated NT-proBNP levels. Therefore, it can be postulated that the use of SGLT2i may exert effective volume reduction effects, which would be more beneficial in HFrEF, but the benefits would be blunted in HFpEF.

However, our study does not necessarily indicate the lack of benefits by SGLT2i in HFpEF, and our findings should be interpreted with caution. Our results would be enough to explain the benefit of SGLT2i in HFrEF in terms of HF prevention, but not sufficient to insist the lack of SGLT2i-induced benefits in HFpEF. At least, given the underlying pathophysiology of HFrEF and HFpEF, we suppose that the benefits of SGLT2i in HFpEF patients would be smaller than those in HFrEF patients. Ongoing clinical trials will provide more concrete evidence for the effects of SGLT2i in HFpEF.

### Limitations

Our study has several limitations. First, our study was a single-center retrospective analysis without pre-specified schedules for echocardiography. Instead, we performed propensity score matching which was successful in terms of comorbidities, medications, and follow-up intervals. Also, the statistical power was acceptable for addressing the changes in echocardiographic parameters in the given sample size. Nonetheless, interpretation of our results needs caution due to the innate limitations of a retrospective study. Second, the diagnosis of HF was based on the information available through medical records, which reflected usual practice. However, the definition of HF in the study was not different from the clinical guidelines [5, 6]. Third, we did not observe comparative benefits of SGLT2i on renal outcomes or glycemic control, which were reported in recent trials [36, 37]. We assume that the lack of renal benefits in our study would be due to the small number of study population and the lack of prespecified schedules for measurements. Finally, we did not investigate the in-depth mechanisms of SGLT2i, and therefore, the mechanistic interpretations of our findings are mainly speculations. While the improvements in LV function by SGLT2i in our study support the cardiovascular benefits of SGLT2i, future studies are required to clarify the biological basis for the effects of SGLT2i on HF.

## Conclusion

In this retrospective cohort study, the use of SGLT2i improved LV systolic and diastolic function, as well as LV geometry, in diabetic patients regardless of the presence of HF. The improvement in LV function was more prominent in those with HFrEF patients compared to those with HFpEF or those without HF. These effects of SGLT2i may contribute to the reduction in HF morbidity and mortality in diabetic patients.

## Supplementary information

**Additional file 1.** Additional tables and figures.

## Data Availability

The datasets used and/or analyzed during the current study are available from the corresponding author on reasonable request.

## Abbreviations

CI: Confidence interval
EF: Ejection fraction
GLS: Global longitudinal strain
HF: Heart failure
HFrEF: Heart failure with reduced ejection fraction
HFpEF: Heart failure with preserved ejection fraction
HR: Hazard ratio
LV: Left ventricle
SGLT2i: Sodium-glucose cotransporter 2 inhibitor
