# Supplementary material for: Different effects of SGLT2 inhibitors according to the presence and types of heart failure in type 2 diabetic patients
Source: Cardiovasc Diabetol. 2020 May 28;19:69. doi: 10.1186/s12933-020-01042-3 (PMC7254690; doi:10.1186/s12933-020-01042-3)
Supplement: Supplementary file 1 — Additional file 1. Additional tables and figures. [file 12933_2020_1042_MOESM1_ESM.docx]

**Supplementary Materials**

**Supplementary Table S1. Baseline characteristics for propensity score matching**

|  | **Global study population**  **(n=8,241)** | **Global population** | | **p-value** |  | **Matched population** | | **p-value** |
| --- | --- | --- | --- | --- | --- | --- | --- | --- |
|  |  | **SGLT2i**  **(n=202)** | **No SGLT2i**  **(n=8,039)** |  |  | **SGLT2i**  **(n=152)** | **No SGLT2i**  **(n=152)** |  |
| **Age (years)** | 67 (57–76) | 63 (53–72) | 68 (57–76) | <0.001 |  | 66 (55–74) | 66 (57–74) | 0.600 |
| **Male sex** | 4,865 (59.0%) | 144 (71.3%) | 4,721 (58.7%) | <0.001 |  | 104 (68.4%) | 95 (62.5%) | 0.335 |
| **Hypertension** | 3,392 (41.2%) | 99 (49.0%) | 3,293 (41.0%) | 0.025 |  | 69 (45.4%) | 79 (52.0%) | 0.302 |
| **Dyslipidemia** | 2,154 (26.1%) | 81 (40.1%) | 2,073 (25.8%) | <0.001 |  | 49 (32.2%) | 52 (34.2%) | 0.808 |
| **Coronary artery disease** | 3,181 (38.6%) | 76 (37.6%) | 3,105 (38.6%) | 0.826 |  | 57 (37.5%) | 54 (35.5%) | 0.812 |
| **Chronic kidney disease** | 701 (8.5%) | 20 (9.9%) | 681 (8.5%) | 0.444 |  | 17 (11.2%) | 19 (12.5%) | 0.859 |
| **Heart failure** | 714 (8.7%) | 74 (36.6%) | 640 (8.0%) | <0.001 |  | 74 (48.7%) | 72 (47.4%) | 0.909 |
| **Atrial fibrillation** | 1,487 (18.0%) | 48 (23.8%) | 1,439 (17.9%) | 0.041 |  | 47 (30.9%) | 48 (31.6%) | 1.000 |
| **Follow-up interval (months)** | 14 (11–19) | 15 (8–21) | 14 (11–19) | <0.001 |  | 13 (8–20) | 12 (8–19) | 0.647 |

The propensity score matching with 1:1 ratio was performed for the patient’s age, sex, hypertension, dyslipidemia, coronary artery disease, heart failure, chronic kidney disease, atrial fibrillation, and interval of echocardiogram, between the eligible patients with SGLT2i treatment (n = 202) and those without SGLT2i treatment (n = 8.039).

Abbreviations: SGLT2i, sodium-glucose cotransporter 2 inhibitor.

**Supplementary Table S2. Detailed information on the use of SGLT2i and cardioprotective medications**

|  | **Total study population**  **(n=304)** | **Group 1**  **No HF, no SGLT2i**  **(n=76)** | **Group 2**  **No HF, SGLT2i (+)**  **(n=78)** | **p-value**  **(groups 1 vs. 2)** | **Group 3**  **HF (+), no SGLT2i**  **(n=76)** | **Group 4**  **HF (+), SGLT2i (+)**  **(n=74)** | **p-value**  **(groups 3 vs. 4)** |
| --- | --- | --- | --- | --- | --- | --- | --- |
| **Use of SGLT2i** |  |  |  |  |  |  |  |
| **Dapagliflozin** | 69 (22.7%) | 0 (0.0%) | 39 (50.0%) | N/A | 0 (0.0%) | 29 (39.2%) | N/A |
| **- Dapagliflozin 5 mg/day** |  |  | 1 (1.3%) |  |  | 0 (0.0%) |  |
| **- Dapagliflozin 10 mg/day** |  |  | 38 (48.7%) |  |  | 29 (39.2%) |  |
| **Empagliflozin** | 83 (27.3%) | 0 (0.0%) | 39 (50.0%) | N/A | 0 (0.0%) | 45 (60.8%) | N/A |
| **- Empagliflozin 10 to 12.5 mg/day** |  |  | 33 (42.3%) |  |  | 39 (52.7%) |  |
| **- Empagliflozin 25 mg/day** |  |  | 6 (7.7%) |  |  | 6 (8.1%) |  |
| **Increased dose during follow-up** |  |  | 4 (5.1%) |  |  | 5 (6.8%) |  |
| **RAS inhibitors** | 268 (88.2%) | 61 (80.3%) | 64 (82.1%) | 0.777 | 71 (93.4%) | 72 (97.3%) | 0.442 |
| **ACEi** | 107 (35.2%) | 20 (26.3%) | 26 (33.3%) | 0.341 | 29 (38.2%) | 32 (43.2%) | 0.526 |
| **Types of ACEi** |  |  |  | 0.786 |  |  | 0.414 |
| - Captopril | 21 (6.9%) | 3 (3.9%) | 5 (6.4%) |  | 4 (5.3%) | 9 (12.2%) |  |
| - Ramipril | 48 (15.8%) | 6 (7.9%) | 8 (10.3%) |  | 17 (22.4%) | 17 (23.0%) |  |
| - Perindopril | 36 (11.8%) | 11 (14.5%) | 12 (15.4%) |  | 7 (9.2%) | 6 (8.1%) |  |
| - Enalapril | 2 (0.7%) | 0 (0.0%) | 1 (1.3%) |  | 1 (1.3%) | 0 (0.0%) |  |
| **Standard dose for HF*** | 27 (8.9%) | 4 (5.3%) | 5 (6.4%) | 0.948 | 9 (11.8%) | 9 (12.2%) | 0.804 |
| **Change in ACEi dose** |  |  |  | 0.447 |  |  | 0.991 |
| - Decreased | 7 (2.3%) | 0 (0.0%) | 1 (1.3%) |  | 3 (3.9%) | 3 (4.1%) |  |
| - No change | 67 (22.0%) | 16 (21.1%) | 17 (21.8%) |  | 16 (21.1%) | 18 (24.3%) |  |
| - Increased | 33 (10.9%) | 4 (5.3%) | 8 (10.3%) |  | 10 (13.2%) | 11 (14.9%) |  |
| **ARB** | 161 (53.0%) | 41 (53.9%) | 38 (48.7%) | 0.516 | 42 (55.3%) | 40 (54.1%) | 0.882 |
| **Types of ARB** |  |  |  | 0.414 |  |  | 0.940 |
| - Losartan | 34 (11.2%) | 7 (9.2%) | 10 (12.8%) |  | 8 (10.5%) | 9 (12.2%) |  |
| - Candesartan | 39 (12.8%) | 11 (14.5%) | 8 (10.3%) |  | 9 (11.8%) | 11 (14.9%) |  |
| - Valsartan | 39 (12.8%) | 6 (7.9%) | 7 (9.0%) |  | 13 (17.1%) | 13 (17.6%) |  |
| - Telmisartan | 19 (6.3%) | 5 (6.6%) | 5 (6.4%) |  | 5 (6.6%) | 4 (5.4%) |  |
| - Irbesartan | 8 (2.6%) | 5 (6.6%) | 0 (0.0%) |  | 2 (2.6%) | 1 (1.4%) |  |
| - Olmesartan | 7 (2.3%) | 3 (3.9%) | 3 (3.8%) |  | 1 (1.3%) | 0 (0.0%) |  |
| - Fimasartan | 12 (3.9%) | 4 (5.3%) | 5 (6.4%) |  | 2 (2.6%) | 1 (1.4%) |  |
| - Azilsartan | 3 (1.0%) | 0 (0.0%) | 0 (0.0%) |  | 2 (2.6%) | 1 (1.4%) |  |
| **Standard dose for HF*** | 39 (12.8%) | 6 (7.9%) | 5 (6.4%) | 0.850 | 16 (21.1%) | 12 (16.2%) | 0.440 |
| **Change in ARB dose** |  |  |  | 0.973 |  |  | 0.999 |
| - Decreased | 13 (4.3%) | 6 (7.9%) | 5 (6.4%) |  | 1 (1.3%) | 1 (1.4%) |  |
| - No change | 99 (32.6%) | 27 (35.5%) | 25 (32.1%) |  | 24 (31.6%) | 23 (31.1%) |  |
| - Increased | 49 (16.1%) | 8 (10.5%) | 8 (10.3%) |  | 17 (22.4%) | 16 (21.6%) |  |
| **Beta-blockers** | 255 (83.9%) | 58 (76.3%) | 61 (78.2%) | 0.780 | 67 (88.2%) | 69 (93.2%) | 0.284 |
| **Types of beta-blockers** |  |  |  | 0.754 |  |  | 0.381 |
| - Carvedilol | 131 (43.1%) | 25 (32.9%) | 25 (32.1%) |  | 38 (50.0%) | 43 (58.1%) |  |
| - Bisoprolol | 89 (29.3%) | 25 (32.9%) | 24 (30.8%) |  | 19 (25.0%) | 21 (30.8%) |  |
| - Nebivolol | 29 (9.5%) | 7 (9.2%) | 9 (11.5%) |  | 8 (10.5%) | 5 (6.8%) |  |
| - Others | 6 (2.0%) | 1 (1.3%) | 3 (3.8%) |  | 2 (2.6%) | 0 (0.0%) |  |
| **Standard dose for HF*** | 30 (9.9%) | 2 (2.6%) | 4 (5.1%) | 0.680 | 11 (14.5%) | 13 (17.6%) | 0.711 |
| **Change in beta-blockers dose** |  |  |  | 0.424 |  |  | 0.273 |
| - Decreased | 23 (7.6%) | 4 (5.3%) | 8 (10.3%) |  | 4 (5.3%) | 7 (9.5%) |  |
| - No change | 126 (41.4%) | 34 (44.7%) | 30 (38.5%) |  | 35 (46.1%) | 27 (36.5%) |  |
| - Increased | 106 (34.9%) | 20 (26.3%) | 23 (29.5%) |  | 28 (36.8%) | 35 (47.3%) |  |
| **MRA** | 103 (33.9%) | 9 (11.8%) | 9 (11.5%) | 0.953 | 40 (52.6%) | 45 (60.8%) | 0.312 |
| **Standard dose for HF*** | 68 (22.4%) | 2 (2.6%) | 4 (5.1%) | 0.620 | 30 (39.5%) | 32 (43.2%) | 0.687 |
| **Change in MRA dose** |  |  |  | 0.681 |  |  | 0.603 |
| - Decreased | 28 (9.2%) | 4 (5.3%) | 4 (5.1%) |  | 8 (10.5%) | 12 (16.2%) |  |
| - No change | 63 (20.7%) | 5 (6.6%) | 5 (6.4%) |  | 25 (32.9%) | 28 (37.8%) |  |
| - Increased | 12 (3.9%) | 0 (0.0%) | 0 (0.0%) |  | 7 (9.2%) | 5 (6.8%) |  |

* Standard doses of ACEi, ARB, beta-blockers, and MRA for HF were determined according to the 2016 ESC Guidelines for the diagnosis and treatment of acute and chronic heart failure.

Abbreviations: HF, heart failure; SGLT2i, sodium-glucose cotransporter 2 inhibitor; RAS, renin-angiotensin system; ACEi, angiotensin converting enzyme inhibitors; ARB, angiotensin receptor blocker; BB, beta-blocker; MRA, mineralocorticoid antagonists.

**Supplementary Table S3. Univariable analysis for the improvement in LV-EF**

|  | **+5% improvement in LV-EF** | | |  | **+10% improvement in LV-EF** | | |
| --- | --- | --- | --- | --- | --- | --- | --- |
|  | **Unadjusted OR** | **95% CI** | **p-value** |  | **Unadjusted OR** | **95% CI** | **p-value** |
| **Age (per +1 year)** | 0.986 | 0.966 – 1.005 | 0.152 |  | 0.976 | 0.952 – 1.001 | 0.056 |
| **Male sex** | 1.040 | 0.633 – 1.710 | 0.877 |  | 0.633 | 0.346 – 1.158 | 0.138 |
| **Hypertension** | 0.605 | 0.375 – 0.975 | 0.039 |  | 0.641 | 0.350 – 1.172 | 0.148 |
| **Dyslipidemia** | 0.615 | 0.366 – 1.033 | 0.066 |  | 0.602 | 0.306 – 1.185 | 0.142 |
| **Coronary artery disease** | 0.957 | 0.586 – 1.563 | 0.860 |  | 0.507 | 0.258 – 0.997 | 0.049 |
| **Chronic kidney disease** | 0.689 | 0.319 – 1.490 | 0.344 |  | 0.940 | 0.371 – 2.387 | 0.897 |
| **Atrial fibrillation** | 1.212 | 0.732 – 2.006 | 0.456 |  | 0.941 | 0.494 – 1.793 | 0.854 |
| **HF** | 2.290 | 1.413 – 3.711 | 0.001 |  | 2.452 | 1.318 – 4.562 | 0.005 |
| **Types of HF*** |  |  |  |  |  |  |  |
| **No HF** | Reference |  |  |  | Reference |  |  |
| **HFpEF (LV-EF** ≥**40%)** | 0.727 | 0.359 – 1.472 | 0.376 |  | 0.659 | 0.234 – 1.858 | 0.431 |
| **HFrEF (LV-EF <40%)** | 4.946 | 2.789 – 8.774 | <0.001 |  | 4.487 | 2.307 – 8.729 | <0.001 |
| **Medications** |  |  |  |  |  |  |  |
| **SGLT2i** | 1.905 | 1.179 – 3.077 | 0.008 |  | 2.017 | 1.092 – 3.726 | 0.025 |
| **- High dose of SGLT2i*** | 0.956 | 0.502 – 1.820 | 0.890 |  | 0.713 | 0.331 – 1.536 | 0.388 |
| **- Increased dose of SGLT2i during follow-up** | 0.672 | 0.162 – 2.795 | 0.585 |  | 0.417 | 0.050 – 3.454 | 0.417 |
| **Statin** | 0.629 | 0.339 – 1.165 | 0.140 |  | 0.466 | 0.230 – 0.944 | 0.034 |
| **Metformin** | 0.923 | 0.503 – 1.696 | 0.797 |  | 1.097 | 0.500 – 2.405 | 0.817 |
| **Sulfonylurea** | 0.577 | 0.357 – 0.932 | 0.025 |  | 0.429 | 0.235 – 0.782 | 0.006 |
| **DPP-4 inhibitors** | 1.210 | 0.722 – 2.029 | 0.470 |  | 0.690 | 0.372 – 1.280 | 0.239 |
| **ACEi** | 1.262 | 0.773 – 2.059 | 0.353 |  | 0.845 | 0.449 – 1.589 | 0.601 |
| **- Standard dose of ACEi for HF** | 1.859 | 0.908 – 3.807 | 0.090 |  | 0.841 | 0.314 – 2.252 | 0.730 |
| **- Increased dose of ACEi during follow-up** | 2.325 | 1.160 – 4.660 | 0.017 |  | 0.888 | 0.348 – 2.268 | 0.805 |
| **ARB** | 0.786 | 0.490 – 1.261 | 0.319 |  | 1.441 | 0.788 – 2.635 | 0.235 |
| **- Standard dose of ARB for HF** | 0.677 | 0.342 – 1.339 | 0.262 |  | 0.545 | 0.225 – 1.320 | 0.178 |
| **- Increased dose of ARB during follow-up** | 1.210 | 0.653 – 2.242 | 0.545 |  | 1.425 | 0.690 – 2.944 | 0.339 |
| **RAS inhibitors** | 0.940 | 0.455 – 1.942 | 0.868 |  | 1.790 | 0.605 – 5.295 | 0.293 |
| **- Standard dose of RAS inhibitors for HF** | 1.652 | 0.973 – 2.084 | 0.063 |  | 0.733 | 0.352 – 1.525 | 0.406 |
| **- Increased dose of RAS inhibitors during follow-up** | 1.244 | 0.712 – 2.174 | 0.442 |  | 1.406 | 0.740 – 2.672 | 0.298 |
| **Beta blockers** | 1.590 | 0.803 – 3.149 | 0.184 |  | 2.676 | 0.919 – 7.794 | 0.071 |
| **- Standard dose of beta blockers for HF** | 1.617 | 0.750 – 3.485 | 0.220 |  | 2.849 | 1.254 – 6.471 | 0.012 |
| **- Increased dose of beta blockers during follow-up** | 1.597 | 1.051 – 2.429 | 0.028 |  | 1.989 | 1.158 – 3.416 | 0.013 |
| **MRA** | 1.896 | 1.159 – 3.102 | 0.011 |  | 2.391 | 1.310 – 4.366 | 0.005 |
| **- Standard dose of MRA for HF** | 0.995 | 0.439 – 2.256 | 0.990 |  | 0.671 | 0.271 – 1.663 | 0.389 |
| **- Increased dose of MRA during follow-up** | 1.150 | 0.604 – 2.190 | 0.670 |  | 1.354 | 0.651 – 2.815 | 0.417 |
| **Diuretics** | 2.499 | 1.501 – 4.161 | <0.001 |  | 3.636 | 1.750 – 7.557 | 0.001 |
| **Laboratory findings at baseline** |  |  |  |  |  |  |  |
| **Hemoglobin (per +1 g/dL)** | 0.994 | 0.885 – 1.115 | 0.917 |  | 1.037 | 0.895 – 1.202 | 0.625 |
| **Serum creatinine (per +1 mg/dL)** | 0.712 | 0.386 – 1.311 | 0.275 |  | 0.684 | 0.303 – 1.543 | 0.360 |
| **Fasting glucose (per +1 mg/dL)** | 1.000 | 0.995 – 1.006 | 0.888 |  | 1.000 | 0.993 – 1.007 | 0.952 |
| **Total cholesterol (per +1 mg/dL)** | 1.005 | 0.999 – 1.010 | 0.120 |  | 1.008 | 1.001 – 1.014 | 0.032 |
| **HbA1c (per +1%)** | 0.958 | 0.816 – 1.126 | 0.605 |  | 0.970 | 0.792 – 1.189 | 0.772 |
| **Echocardiographic findings at baseline** |  |  |  |  |  |  |  |
| **LV-EDD (per +1mm)** | 1.052 | 1.022 – 1.082 | <0.001 |  | 1.059 | 1.024 – 1.096 | 0.001 |
| **LV-ESD (per +1mm)** | 1.050 | 1.027 – 1.074 | <0.001 |  | 1.058 | 1.030 – 1.086 | <0.001 |
| **LV-EDV (per +1mL)** | 1.011 | 1.006 – 1.016 | <0.001 |  | 1.012 | 1.006 – 1.018 | <0.001 |
| **LV-ESV (per +1mL)** | 1.015 | 1.009 – 1.021 | <0.001 |  | 1.015 | 1.009 – 1.021 | <0.001 |
| **LV-EF (per +1%)** | 0.944 | 0.928 – 0.960 | <0.001 |  | 0.936 | 0.916 – 0.956 | <0.001 |
| **LV-MI (per +1g/m^2^)** | 1.011 | 1.003 – 1.018 | 0.005 |  | 1.015 | 1.006 – 1.024 | 0.001 |
| **LAVI (per +1mL/m^2^)** | 1.011 | 1.001 – 1.021 | 0.032 |  | 1.010 | 0.999 – 1.021 | 0.065 |
| **PASP (per +1 mmHg)** | 1.034 | 1.013 – 1.055 | 0.001 |  | 1.023 | 1.001 – 1.045 | 0.040 |
| **E velocity (per +1 m/sec)** | 2.404 | .0988 – 5.850 | 0.053 |  | 2.168 | 0.763 – 6.155 | 0.146 |
| **Mitral annular e’ velocity (per +1 cm/sec)** | 0.988 | 0.876 – 1.113 | 0.840 |  | 0.910 | 0.780 – 1.061 | 0.229 |
| **Mitral annular s’ velocity (per +1 cm/sec)** | 0.971 | 0.902 – 1.046 | 0.440 |  | 0.778 | 0.653 – 0.927 | 0.005 |
| **Mitral E/e’ ratio** | 1.015 | 0.988 – 1.043 | 0.273 |  | 1.025 | 0.993 – 1.058 | 0.126 |
| **LV-GLS (per +1%)** | 0.878 | 0.826 – 0.934 | <0.001 |  | 0.856 | 0.792 – 0.924 | <0.001 |

Univariable logistic regression analyses were performed for the +5% improvement of LV-EF (left column) and for the +10% improvement of LV-EF (right column) on the follow-up echocardiogram.

* Analysis for the types of HF was performed using the patients without HF as a reference.

Abbreviations: OR, odds ratio; CI, confidence interval; HF, heart failure; HFpEF, heart failure with preserved ejection fraction; HFrEF, heart failure with reduced ejection fraction; SGLT2i, sodium-glucose cotransporter 2 inhibitor; DPP-4, dipeptidylpeptidase-4; RAS, renin-angiotensin system; MRA, mineralocorticoid antagonist; LV, left ventricular; EDV, end-diastolic volume; ESV, end-systolic volume; EDD, end-diastolic dimension; ESD, end-systolic dimension; EF, ejection fraction; MI, mass index; LAVI, left atrial volume index; PASP, pulmonary artery systolic pressure; GLS, global longitudinal strain.

**Supplementary Figure S1. Changes in LV geometry and systolic function**


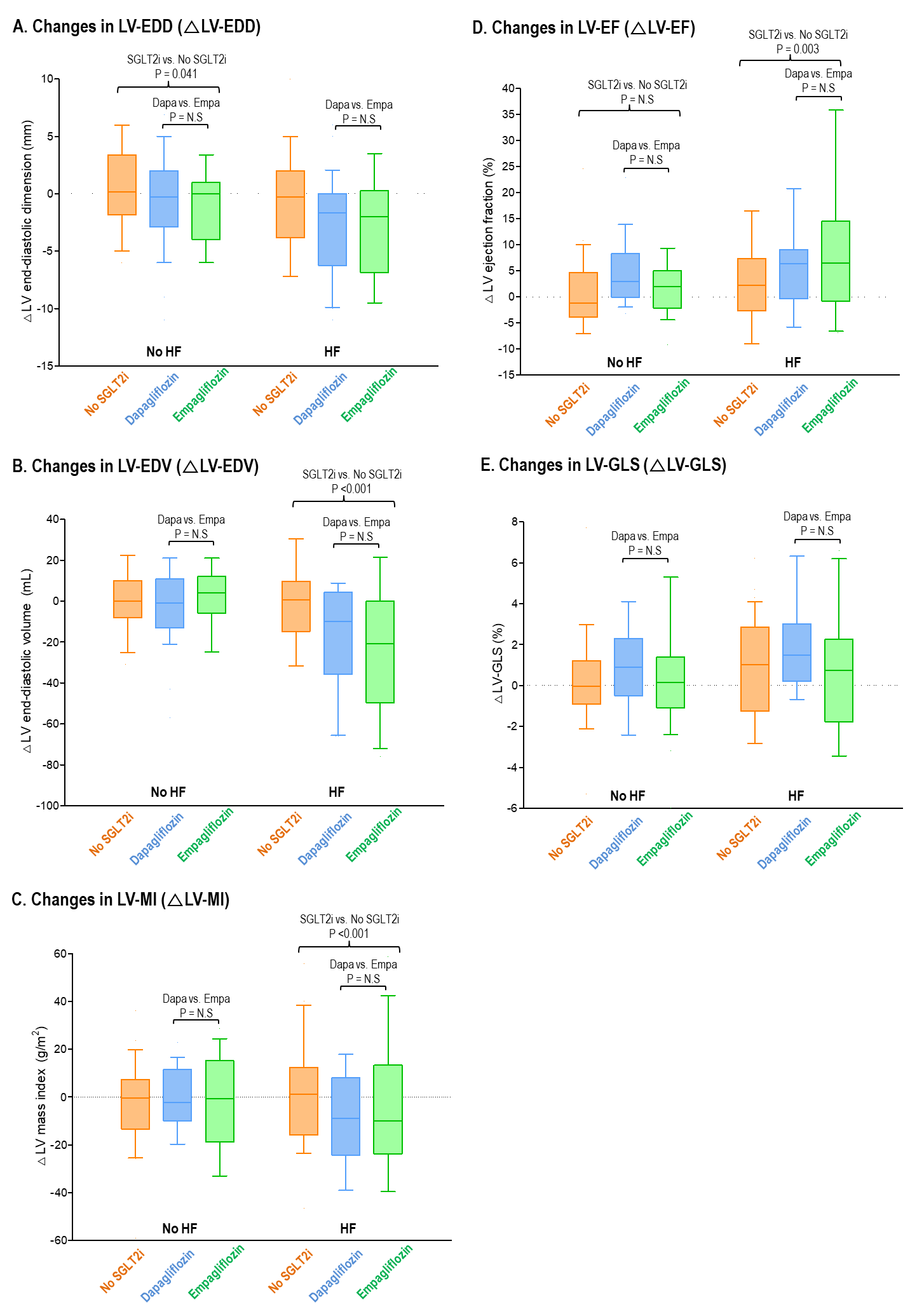


Changes in LV-EDD, LV-EDV, LV-MI, LV-EF, and LV-GLS are compared between the patients without SGLT2i (orange), those on dapagliflozin (blue), and those on empagliflozin (green), according to the presence of HF.

**Supplementary Figure S2. Changes in LV diastolic function**


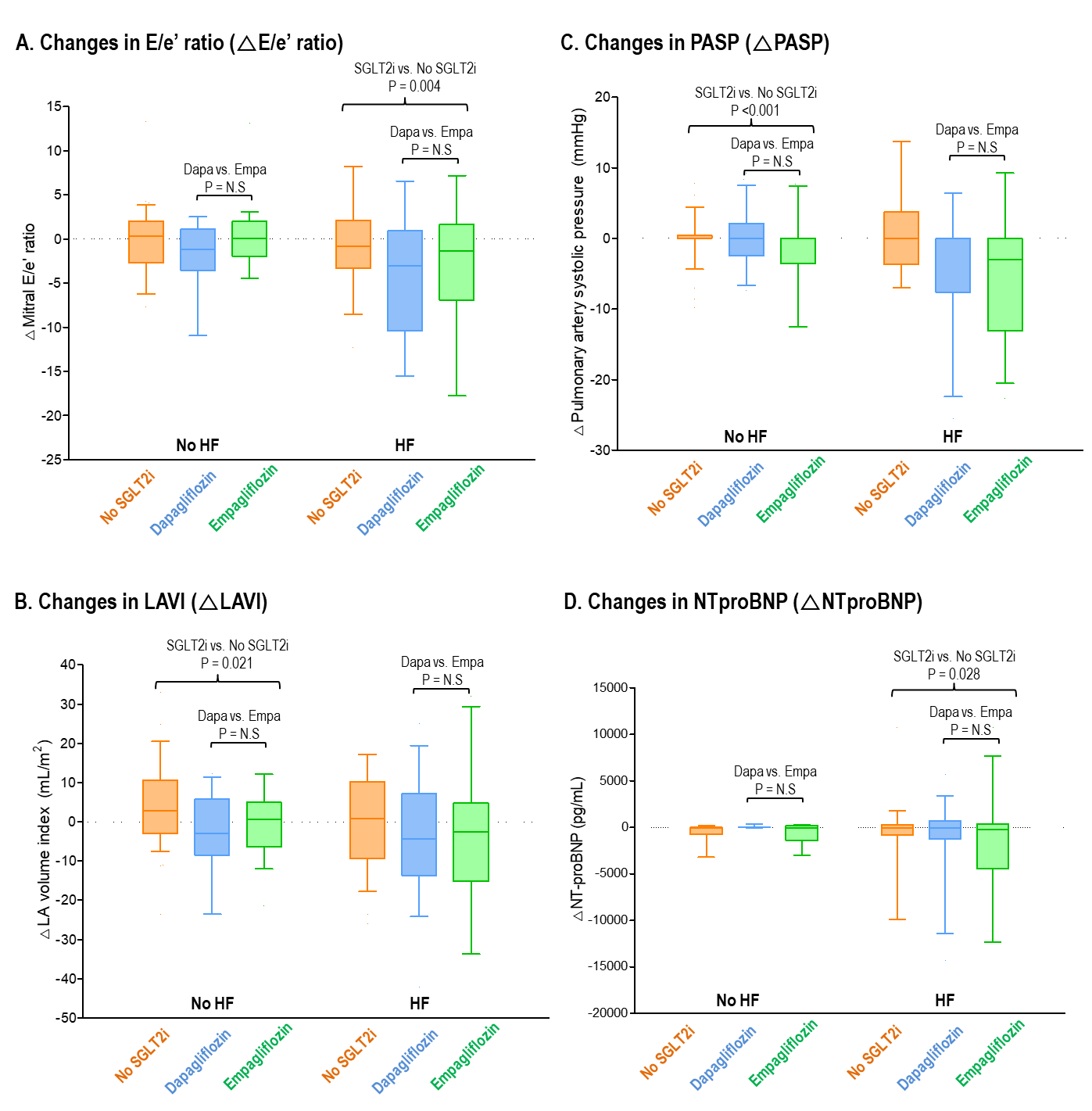


Changes in mitral E/e’ ratio, LAVI, PASP, and NT-proBNP are compared between the patients without SGLT2i (orange), those on dapagliflozin (blue), and those on empagliflozin (green), according to the presence of HF.
